# The benefits and limitations of animal models for translational research in cartilage repair

**DOI:** 10.1186/s40634-015-0037-x

**Published:** 2016-01-06

**Authors:** Conor J. Moran, Ashwanth Ramesh, Pieter A. J. Brama, John M. O’Byrne, Fergal J. O’Brien, Tanya J. Levingstone

**Affiliations:** Tissue Engineering Research Group, Department of Anatomy, Royal College of Surgeons in Ireland, 123 St. Stephen’s Green, Dublin 2, Ireland; Trinity Centre for Bioengineering, Trinity College Dublin, Dublin 2, Ireland; Advanced Materials and Bioengineering Research (AMBER) Centre, RCSI & TCD, Dublin, Ireland; Section of Veterinary Clinical Sciences, School of Veterinary Medicine, University College Dublin, Dublin, Ireland; Cappagh National Orthopaedic Hospital, Finglas, Dublin 11, Ireland

**Keywords:** Tissue engineering, Collagen, In vivo, Osteochondral, Cartilage

## Abstract

Much research is currently ongoing into new therapies for cartilage defect repair with new biomaterials frequently appearing which purport to have significant regenerative capacity. These biomaterials may be classified as medical devices, and as such must undergo rigorous testing before they are implanted in humans. A large part of this testing involves in vitro trials and biomechanical testing. However, in order to bridge the gap between the lab and the clinic, in vivo preclinical trials are required, and usually demanded by regulatory approval bodies. This review examines the in vivo models in current use for cartilage defect repair testing and the relevance of each in the context of generated results and applicability to bringing the device to clinical practice. Some of the preclinical models currently used include murine, leporine, ovine, caprine, porcine, canine, and equine models. Each of these has advantages and disadvantages in terms of animal husbandry, cartilage thickness, joint biomechanics and ethical and licencing issues. This review will examine the strengths and weaknesses of the various animal models currently in use in preclinical studies of cartilage repair.

## Introduction

Cartilage defects have long caused significant morbidity for patients and present difficulty for surgeons attempting repair. Due to the avascular nature of the chondral surface and the specialised rigid extracellular matrix with a low cell density this tissue rarely regenerates by itself (Mankin 1982). There is significant ongoing research focused on halting the propagation of these defects and the eventual requirement for joint replacement. Current clinical procedures include bone marrow stimulation techniques, cartilage plug transplant, and expanded autologous chondrocyte implantation (Camp et al. 2014; Steadman et al. 2003; Brittberg et al. 1994; Peterson et al. 2003; Hangody et al. 2001). In recent years, the focus has moved to bioengineered materials and cell-seeded bioengineered scaffolds (Levingstone et al. 2014; Almeida et al. 2015; Hunziker et al. 2015). While in vitro testing and biomechanical analysis of biomaterials can provide much information about the safety, efficacy and potential for repair of these new biomaterials, in order to truly assess their regenerative capabilities, and the immune response associated with implantation, the use of animal models is required. Furthermore, regulatory bodies require in vivo animal studies to be carried out before new devices can be translated into clinical practice (Chu 2001; Hoemann et al. 2011; Hurtig et al. 2011). When carrying out animal studies the principles of the three R’s; Reduction, Replacement and Refinement must applied (Russell & Burch 1959). The number of animals used should be reduced to the minimum required to achieve a valid statistically significant result. Wherever possible the use of animals should be replaced by other means, such as computer simulation, cadaveric, or in vitro testing, and the experiment must refined or altered in any way possible so as to decrease potential for suffering for all involved animals.

## Review

### General considerations when selecting an appropriate animal model for the assessment of biomaterials strategies for cartilage repair

A range of factors require consideration when selecting an appropriate animal model for the assessment of biomaterial strategies for cartilage repair. Firstly a decision on small or large animal model is required is necessary: small animal models include rodents such as mice, rats and rabbits; while large animal models include dogs, goats, sheep, pigs and horses. Each has advantages and disadvantages, in the assessment of the clinical potential of new materials the model that most closely representing human anatomy and physiology of healing should be considered. Factors requiring consideration include, the size of the joint, the cartilage thickness, the depth and critical size of the defect (critical size implies a defect which will not heal spontaneously without any intervention), the age of skeletal maturity (better results in young patients regardless of treatment type), load distribution of the stifle, affordability and ease of animal handling. (Hunziker et al. 2002) Additionally, the defect position is closely related to its loading conditions and is therefore an important consideration. Although clinically most defects occur on the weight bearing medial condyle of the femur, many animal studies choose a partial-weight bearing site such as the trochlea, to improve the retention of the scaffold and cells implanted. (Hoemann et al. 2011). While many studies report improved cartilage repair in defects positioned in the trochlear over the femoral condyle (Orth et al. 2013a), conflicting results, where less defect repair occurs in the trochlea compared to the medial condyle have also been reported (Hoemann et al. 2011; Hoemann et al. 2005). In the clinical scenario most operators will follow surgery with a 4–6 week period of partial-weight bearing with full range of motion in the joint, slowly working up to full weight bearing (Vogt et al. 2013) even though short term studies have shown an accelerated rehab protocol can reduce pain and increase function (Vogt et al. 2013; Ebert et al. 2008) In the preclinical in vivo model weight bearing can be controlled by using external fixators and casts on the animals (Kojima et al. 2014; Roth et al. 1988). Confining animals to pens for the initial post-operative period has been shown to be effective in reducing post-operative joint load (Etterlin et al. 2014). Many studies favour uni-lateral (one treatment per animal) models (Marmotti et al. 2012; Nixon et al. 2015), however, bi-lateral models offer the advantages of allowing direct comparison of a treatment to a control within the same animal, thus counteracting the effect of host factors such as age, sex, weight, tissue characteristics, physical activity, or hormonal status and enabling a reduction in animal numbers (Orth et al. 2013b). Consideration of post-operative mobilisation is important in model selection and bi-lateral models are unsuitable when unloading of the treated joint is necessary or when gait analyses are to be performed. Guidelines have been set out in ASTM F 2451-05 for animal models suitable for the assessment of cartilage repair (Steadman et al. 2003). A comparison of the properties of various animal models used for the assessment of biomaterial approaches to cartilage defect repair is present in Table 1.

**Table 1:** A comparison of various models used in preclinical models for the assessment of biomaterial strategies for cartilage defect repair to the human knee joint

Species	Breed	Age of skeletal maturity	Adult weight	Cartilage thickness	Calcified cartilage layer thickness	Bone plate thickness	Critical size defect	References
Human		18–22 years	60–90 kg	2.4–2.6 mm	0.1 mm	0.2–0.5 mm	10 mm	Chevrier (Chevrier et al. 2015), ASTM (Dahlin et al. 2014)
Rabbit	New Zealand White	9 months	3–4 kg	0.16–0.75 mm	0.1–0.15 mm	0.4–0.5 mm	3 mm	Chevrier (Chevrier et al. 2015). ASTM (Dahlin et al. 2014)
Dog	Mongrel, Beagle	1–2 years	15–30 kg	0.95–1.3 mm	-	-	4 mm	Ahern (Ahern et al. 2009), ASTM (Dahlin et al. 2014)
Mini-pig	Gottingen Mini-pig, Yucatan, Lee-sung	10 months–1 year	20–40 kg	1.5 mm–2.0 mm	-	-	6 mm	Ahern (Ahern et al. 2009), ASTM (Dahlin et al. 2014)
Pig	Large White	2 years	250 kg	1.5 mm-2.0 mm	-	-	6 mm	Ahern (Ahern et al. 2009), ASTM (Dahlin et al. 2014)
Goat	Spanish, Dairy, Boer Cross, Saanan	2–3 years	40–70 kg	0.8–2 mm	0.2 mm	0.3 mm	6 mm	Patil (Patil et al. 2014), ASTM (Dahlin et al. 2014), Frisbie (Frisbie et al. 2009),
Sheep	Suffolk, Texel	2–3 years	35–80 kg	0.7–1.7 mm	0.2 mm	0.7 mm	7 mm	Chevrier (Chevrier et al. 2015), ASTM (Dahlin et al. 2014)
Horse	Mixed, Thoroughbred, Quarter Horse	2–4 years	500–600 kg	2.0–3.0 mm	0.2 mm	0.7 mm	9 mm	Chevrier (Chevrier et al. 2015), ASTM (Dahlin et al. 2014)

## Small animal models

### Rodent models

Small animal models can be very useful to give information about the residence time of an implant, and also to determine the type of repair tissue formed (ASTM F2451-05 2010). The availability of athymic, transgenic and knockout strains of both rats and mice means these models can be used to assess a multitude of factors including the use of strains of mice (DBA/1) in which osteoarthritis occurs spontaneously (Nordling et al. 1992) and athymic strains which can be used to assess allogenic and xenogeneic cells and tissues (Chu et al. 2010a). Rodents, such as mice and rats, have the advantage of being purpose bred to reduce biological variation, while being affordable, and easy to breed and maintain in-house. They thus act as a good bridge between in vitro and in vivo experiments to provide proof of concept data; however, their joints are small with very thin cartilage consisting of only a few cell layers (Chu et al. 2010a). Rodent models can provide useful subcutaneous models and intramuscular models for the assessment of the degradation rate and safety profile of biomaterials and implants generally 6–8 weeks in duration (Chu et al. 2010a). Rat models are not frequently used in the assessment of chondral defect repair due to the thinness of the cartilage layer (Gelse et al. 2003; Kuroda et al. 2006). However, a study by Choi et al. reports the use of chondral defects 1 mm in diameter and 0.15 mm deep on the femoral trochlear groove for assessment of growth factor releasing hydrogels (Choi et al. 2015). Additionally osteochondral defects of 1.5 mm (Dahlin et al. 2014) and 2 mm (Chung et al. 2014) diameter on the femoral condyle have been used for the assessment of biomaterial strategies (Singh et al. 2013). These models have limited potential in the determination of the clinical potential of new biomaterials as repair processes that are successful in restoring such small diameter defects may not be applicable in larger defect (Chu et al. 2010a). Rodents also have open growth plates throughout their maturity (Libbin & Rivera 1989) and therefore may have the increased intrinsic healing capacity of a juvenile. Additionally, the gait pattern and biomechanical loading environment in rodents varies significantly to that in humans. Rodents are therefore very limited in their potential to be used as a translational model for cartilage surface repair in humans (Chu et al. 2010a).

### Rabbit model

**Fig. 1:**
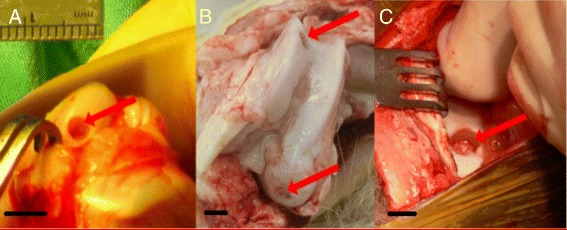
Macroscopic image of distal femur of (**a**) rabbit, (**b**) goat and (**c**) horse showing (**a**) 3 mm, (**b**) 6 mm and (**c**) 9 mm defects created by drilling. This demonstrates the significant difference in the size of the joints involved and the size of the defects that can be created using these models. (Scale bar = 5 mm)

**Fig. 2:**
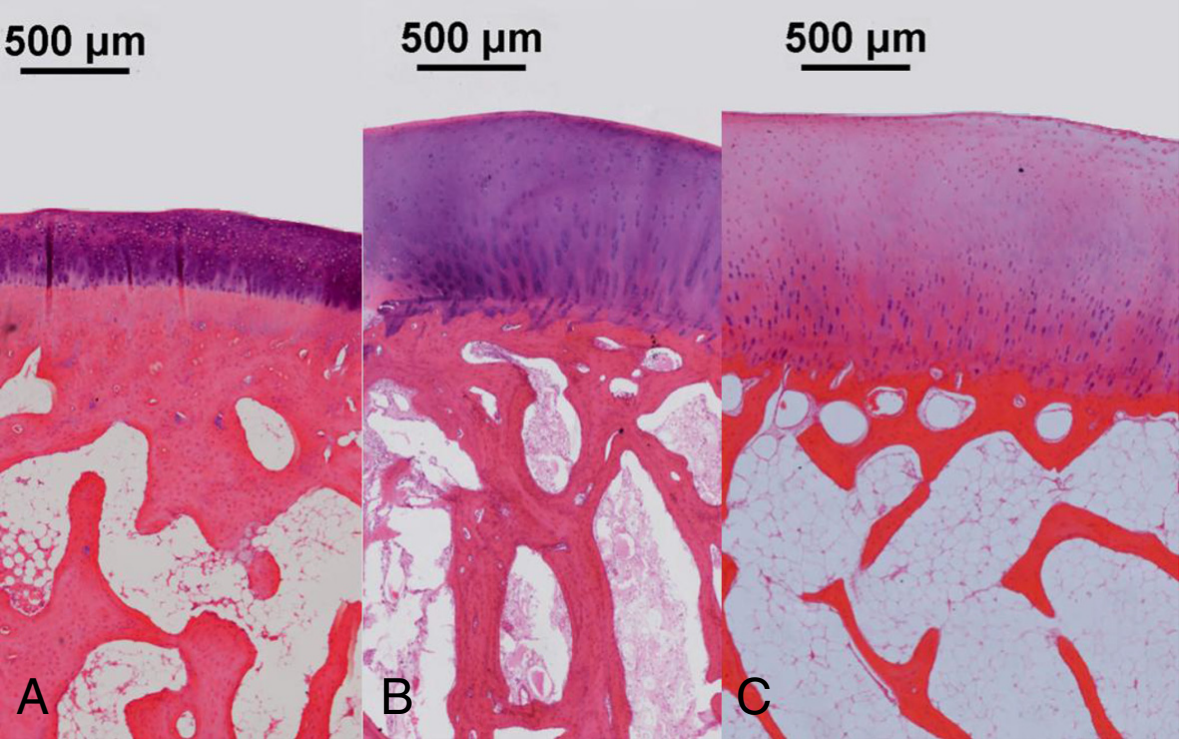
H&E stained histology specimens of the distal femur of (**a**) rabbit (**b**) goat and (**c**) horse. These images demonstrate the histological similarity between the different models, but also the vast differences in the thickness of the cartilage at the joint surface. The chondrocyte distribution differences are also evident, with the rabbit cartilage being much more densely packed with chondrocytes than either goat or horse which could explain some better intrinsic healing of cartilage defects in rabbits

The rabbit model provides a more suitable small animal model for the assessment of cartilage repair as they have larger joints and are a good size for easy surgical procedures and specimen handling with a cartilage thickness of 0.25 mm–0.75 mm (Table 1) (Fig. 1.) (ASTM F2451–05 2010). Rabbits have been widely used for the assessment of cartilage repair in studies lasting up to 16 weeks, although some 1 year rabbit studies have been performed (Maruyama 1979; Brittberg et al. 1997; Fragonas et al. 2000; Yanai et al. 2005; Funayama et al. 2008; Luengo Gimeno et al. 2006; Chu et al. 1997; Levingstone et al. 2015). Rabbits offer many advantages as they are cost effective, easy to handle and to house. The femoral condyle is the most often used defect site for weight bearing models, especially those located inferioposteriorly (An & Freidman 1999). However, a comprehensive biokinematic study of rabbit gait pattern during hopping performed by Gushue et al. (Gushue et al. 2005) noted that, due to the wide variety of landing patterns of the hind limb during hopping, there are increased forces in the lateral tibiofemoral joint with a mean of 262.3 % body weight going through the medial side, and 303.8 % body weight in the lateral joint (Gushue et al. 2005). This correlates with previous studies showing increased bone mineral density at the lateral tibial plateau and balanced subchondral tissue volume (Messner et al. 2001; Wei et al. 1998). Additionally, the rabbit hind limbs are kept primarily in a fully flexed position as opposed to human weight bearing which is primarily on a knee locked in extension (Madry et al. 2015). Thus due to the differing biomechanics, caution is therefore advised when comparing results from rabbit studies to humans. Intercondylar groove defects have been used as partial weight bearing defects. (ASTM F2451–05 2010; Chu et al. 1997). Greater rates of repair may occur in rabbit articular cartilage models compared to other species due to higher metabolic activity and density of pluripotent stem cells near the defect site (Fig. 2) (ASTM F2451–05 2010). In addition, while the size of chondrocytes in human and rabbit articular cartilage do not differ significantly from each other (Hunziker 1999), the overall cell volume density is approximately 1.7 % in cartilage from the human medial femoral condyle (MFC) as opposed to 12.2 % in the adult rabbit. These amount to cell densities of 1800 and 7500 per mm^3^ in humans and rabbits respectively (Hunziker 1999). The low cellularity of human hyaline cartilage thus contributes to the poor levels of repair observed while the increased density of chondrocytes in rabbits means there are more cells abutting the defect site for repair. The bone mineral density in the rabbit medial femoral condyle (MFC) is reported to be similar to that in humans at the bone plate (1.19 g/cm^3^ and 1.17 respectively g/cm^3^) but at a depth of 3 mm was 0.65 g/cm^3^ compared to 0.36 g/cm^3^ in humans. (Chevrier et al. 2015) Bone volume fraction was 58 ± 10 % in the rabbit MFC compared to 33 ± 13 % in humans (Chevrier et al. 2015). Rabbit stifle joints have different load characteristics and cartilage thickness compared to humans, making it difficult to investigate translation potential in this model.

## Large animal models

Short (8–12 weeks) studies can be used to provide information regarding the biocompatibility, early cellular responsiveness and persistence and condition of the implant within the defect. Longer studies (6–12 months) are necessary to gain confidence in extent of success in the repair and regeneration of articular cartilage, including interface with adjacent cartilage and subchondral bone as well as the opposing articular surface (ASTM F2451–05 2010). A range of large animal models suitable for the assessment of cartilage repair have been investigated, including dogs (Engkvist 1979; Igarashi et al. 2012; Breinan et al. 2000), pigs (Hunziker et al. 2001; Lohan et al. 2013; Boopalan et al. 2011; Klein et al. 2009; Christensen et al. 2015), sheep (Kon et al. 2010a; Milano et al. 2010; Erggelet et al. 2009), goats (Getgood et al. 2012; Jurgens et al. 2013; Lu et al. 2006; Wang et al. 2007) and horses (Kon et al. 2010b; Frisbie et al. 2009; Hendrickson et al. 1994). When using any large animal model it is important to determine where on the joint the defect should be created based on the biomechanics of the joint. In humans, most cartilage defects occur on the weight bearing medial condyle this is thus the most common defect site used in cartilage repair studies (An & Freidman 1999). If using this defect type, the following should be considered in order to meet ASTM F2451-05 (ASTM F2451–05 2010).

1. The defect size should not exceed 15 to 20 % of the articulating surface or 50–60 % of the condylar width.
2. Due to the convex curvature of the defect sites the defect can differ from the centre to the margins.
3. It is necessary to consider the impact of articulation with both the meniscus and the tibial plateau.

### Canine model

Dogs suffer from many of the cartilage pathologies found in humans like osteochondritis dissecans and osteoarthritis (Shortkroff et al. 1996) and veterinary surgeons regularly perform arthroscopies on canine stifles. As such, canine models are considered to be a good choice for cartilage repair studies (An & Freidman 1999). They accept rehabilitation regimens, cope well with immobilising the joint, and can be trained to walk on treadmills, and can co-operate in swimming and controlled weight bearing rehabilitation (Hurtig et al. 2011). The cartilage thickness, however, is significantly thinner than human cartilage even in medium to large dogs (range: 0.95–1.3 mm) (Table 1) and the reported diameter of critical size defects, at 4 mm, is considerably small even in the largest dogs (Ahern et al. 2009). Anatomical differences are present between canine and human knee joints with the existence of an extra intra-capsular, extra-articular, lateral long digital extensor tendon (LDET) which originates just inferior to the lateral edge of the patellar groove in the canine joint. Its function is dorsiflexion of the forefoot during knee flexion and is found in many quadrupeds (Proffen et al. 2012). Therefore, although canines deal well with the perioperative regimen, the canine knee joint does not model the human knee joint very closely Due to their longstanding status as companion and family pet, ethical issues also prevent their widespread use. In the UK and Ireland cats, dogs, horses, non-human primates and endangered species require a special justification for use to show no other species is suitable for the specified programme of work. It is therefore easier to get ethical approval for agricultural animals such as pigs, goats and sheep than canine models (An & Freidman 1999; Animals (Scientific Procedures) Act. Sect. 14 1986; Wolfensohn & Lloyd 2003).

### Porcine model

Pigs (porcine) models are advantageous in the terms of their joint size, joint loading mechanics, weight (an adult sow can weigh up to 250 kg) (Wolfensohn & Lloyd 2003), lack of spontaneous healing of any significant defects, bone trabecular thickness and the arrangement of collagen network which resemble a human joint (Chu et al. 2010a). Nevertheless, these are large animals and require specialised husbandry and can be expensive to maintain in a research facility. They do not reach skeletal maturity until approximately two years and although researchers can utilise stock from commercial companies and farmers, most pigs will be slaughtered around the age of 6 months and therefore sourcing of skeletally mature animals of a similar age is often difficult. The alternative use of mature breeding animals makes sourcing of sufficient numbers, good health status and uniform age difficult because these animals are usually only replaced because of health problems on a one by one basis.

Mini-pigs are significantly smaller than full sized swine weighing roughly 40–70 kg as adults (Christensen et al. 2015; Wolfensohn & Lloyd 2003) and can thus provide some of the advantages of the pig model while overcoming some of the limitations (Schneider et al. 2011; Ebihara et al. 2012). The physiological parameters, such as blood count, blood clotting, electrolytes and liver enzymes, have been shown to be similar to values for humans (Chu et al. 2010a). A range of breeds of mini-pig have been utilised in the assessment of biomaterials for cartilage repair including Yucatan (Fisher et al. 2015), Gottingen (Schneider et al. 2011) and Lee-sung (Jiang et al. 2007) mini-pigs. Immature Yucatan mini-pigs are reported to have cartilage of 1–2 mm in thickness on the medial femoral condyle (Fisher et al. 2015). Histomorphometric analysis of peripheral bone in Gottingen mini-pigs has shown the bone apposition rate and trabecular thickness to be similar to human bone (Chu et al. 2010a), which is a significant factor when measuring the inflammatory response and toxicity of any implanted biomaterials. Mini-pigs of a defined type and known health status can be sourced from specialist laboratory suppliers but they are not skeletally mature until they reach 18–22 months of age (Chu et al. 2010a) and require specialist housing including specialised slatted flooring separate from a dry bedding area. Many studies thus use immature mini-pigs that have not reached skeletal maturity and thus the data reported has limited clinically relevance. Pigs will also turn any pasture into a mud bath by rooting for food and so can be expensive to maintain in a long term study (Wolfensohn & Lloyd 2003).

### Caprine model

**Fig. 3:**
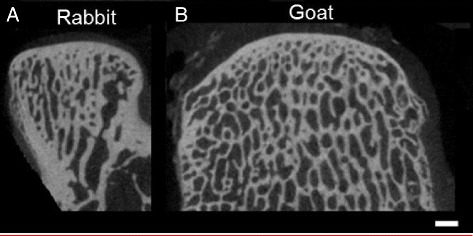
2D micro-CT sections from rabbit (**a**) and goat (**b**) medial femoral condyles. These images demonstrate the similarity between the both models, with similar bone plate thickness and trabecular thickness in both cases. (Scale = 2 mm)

Two of the most commonly used models in research are ruminants, these are the caprine and ovine models. Goats are among the earliest animals domesticated by humans. They are farmed throughout the world and are used for a variety of products, including milk, meat and coat fibres (mohair and cashmere). They are, as a result, relatively easy to obtain when skeletally mature. The caprine (goat) femoral condyle and trochlear defect models have been used successfully for evaluation of new implants for treatment of partial thickness and osteochondral defects (Fig. 1) (Klein et al. 2009; Jurgens et al. 2013; Nukavarapu & Dorcemus 2013). Such models offer the advantages of joint size, cartilage thickness (although the ASTM F 2451–05 reports thicknesses of 1.5–2.0 mm, there are reports in the literature ranging from 0.8 mm (ASTM F2451–05 2010; Chu et al. 2010a; Brehm et al. 2006)) (Table 1), critical defect size (6 mm is the most commonly reported and will not spontaneously heal at 6 months) (Getgood et al. 2012; Ahern et al. 2009; Shahgaldi 1998; Jackson et al. 2001) and proportion of cartilage to bone and subchondral bone thickness being close to humans (Ahern et al. 2009; Jackson et al. 2001; Chu et al. 2010b). Subchondral bone trabecular structure in goats is similar to that in humans (Fig. 3) and bone mineral density has been reported to be 0.67 g/cm^3^ (Gollehon et al. 1987). The caprine stifle joint, like the human knee consists of tibiofemoral and patellofemoral articulations. In a direct comparative study of the stifle joint of cows, sheep, goats, dogs, pigs, and rabbits the goat stifle was found to have the closest anatomy to the human knee (Proffen et al. 2012). However, the femur has a deep long trochlear groove with prominent medial and lateral ridges. The femoral condyles are also distinct with a large intercondylar notch. The tibial plateau is convex and sloped posterolaterally with a prominent fibular styloid laterally roughly correlating to the fibular head and styloid process in humans (LaPrade et al. 2006). Additionally, the soft tissue structures which prevent abnormal joint movement specifically in the lateral compartment of the goat knee are similar to those in the human knee joint; these include lateral collateral ligament (LCL) complex, popliteus tendon and popliteofibular ligament. These structures act as primary stabilisers to prevent abnormal varus and tibial external rotation while also acting as secondary stabilisers, preventing anterior and posterior translation of the knee (Gollehon et al. 1987). Articular congruity at the tibiofemoral joint is poor in both humans and goats due to the convex surface of the tibial plateau, but goats have significantly thicker menisci, which contribute to increasing congruity between goats and humans (Patil et al. 2014). In the human knee flexion is limited to less than 30° in normal walking and stance phase however the goat stifle joint is flexed between 50°and 70° (Patil et al. 2014) meaning contact areas are different and must be considered.

Caprine cartilage is slightly thinner than the human distal femoral cartilage (Hoemann et al. 2011; ASTM F2451–05 2010) and the stiffness and elastic modulus of the caprine cartilage has been found to be greater than human articular cartilage (Patil et al. 2014). However in depth biomechanical analysis of the differences between the goat stifle joint and the human knee joint has shown that when the joint is loaded under conditions representing normal walking (ranging from 25 % body weight to 200 % body weight), most of the peak contact pressures in goat knees were comparable to those generated in human knees. (Patil et al. 2014) It did show, however the peak contact pressures in the caprine tibiofemoral joint at peak flexion are higher than normal human tibiofemoral contact pressure in the normal walking cycle, under twice body weight at peak flexion of normal walking (70°) goats reach a contact pressure of 12.57 MPa whereas in normal walking humans only flex to 30° and reach 4.93 MPa (Patil et al. 2014). Aside from this peak, the biomechanics and contact pressures of the goat walking cycle are broadly similar to the human equivalent. Goats are also relatively inexpensive to maintain, easy to handle, and the cartilage thickness allows for the creation of both chondral and osteochondral defects (Chu et al. 2010a). If adequate facilities are available to house them, then this model is feasible to conduct large animal studies to evaluate biological responses, durability, toxicology, lesion size and location analogous to human studies (Cook et al. 2014). Caprine models thus represent a good option for in vivo assessment of chondral and osteochondral defect repair.

### Ovine model

The sheep (ovine) model is one commonly used for in vivo trials of materials for cartilage repair (Kon et al. 2010a; Milano et al. 2010; Guo et al. 2004). Weighing between 35 and 80 kg when skeletally mature at 2–3 years they transmit a scientifically relevant amount of weight through its tibiofemoral joint (Wolfensohn & Lloyd 2003). They have a cartilage thickness of roughly 0.4–1.7 mm at the medial condyle (ASTM F2451–05 2010; Ahern et al. 2009). The sheep stifle joint is a diarthrodal joint with four separate articulations: femoropatellar, femorotibial, femorofibular and tibiofibular. Like the goat, the sheep has a long trochlear groove formed by prominent medial and lateral trochlear ridges The femorotibial joint has a range of motion from 72+/−3° in full flexion to 145 +/− 5° in full extension (Allen et al. 1998). A large amount of research can be done using the ovine stifle joint as it has very similar cruciate ligaments to humans and large menisci as well as a similar LCL complex, popliteus tendon and popliteofibular ligament and can therefore be used in surgical training as well as device development (Madry et al. 2015; Allen et al. 1998). This also means second look arthroscopy can be performed by a skilled arthroscopist to review integration (Ahern et al. 2009).

Sheep are also readily available as they are commonly bred in agriculture and are relatively placid, tolerate stifle surgery well and are easily housed and maintained. They have, however, been reported to have a very variable articular cartilage thickness, between 0.4 and 1.7 mm on the MFC (Ahern et al. 2009), this variability can cause issues with study design and results. Bone mineral density in sheep MFC has been shown to be similar to that in humans, reportedly 1.19 g/cm^3^. However at a depth of 3 mm the bone mineral density is reportedly 0.67 g/cm^3^ compared to 0.36 g/cm^3^ in humans. (Chevrier et al. 2015) The bone volume fraction in the MFC is reportedly higher in sheep than humans at 3 mm below the bone surface (42 ± 4 % and 33 ± 13 % respectively) (Chevrier et al. 2015). Contact pressures generated in sheep are largely comparable to those in humans, although while humans can reach a mean peak contact force of 5.4 times body weight ascending stairs (Taylor et al. 2004) the maximum in vivo contact force measured in sheep is 2.25 times body weight (Patil et al. 2014; Taylor et al. 2006).

Techniques that expose the subchondral bone such as the implantation of osteochondral repair scaffolds or osteochondral allografts, may induce subchondral bone cyst formation either through fluid intrusion or bony contusion. Increased formation of cysts in the subchondral bone have been reported in both goats and sheep models (von Rechenberg et al. 2003; Orth et al. 2012). This cyst formation can hamper subchondral bone repair (von Rechenberg et al. 2003; Pallante-Kichura et al. 2013). Other disadvantages include extra fat pad that can obscure the joint and the more labour intensive husbandry practices required for animal handling (Wolfensohn & Lloyd 2003) and the acquisition of animals from an agricultural background instead of bred for purpose, meaning their health status and genetic background will be less uniform. The use of goats and sheep in research is much less common than the use of rodents and thus specialised commercial products, such as antibodies, available to researchers utilising rodent models are not readily available for ruminants.

### Equine model

**Fig. 4:**
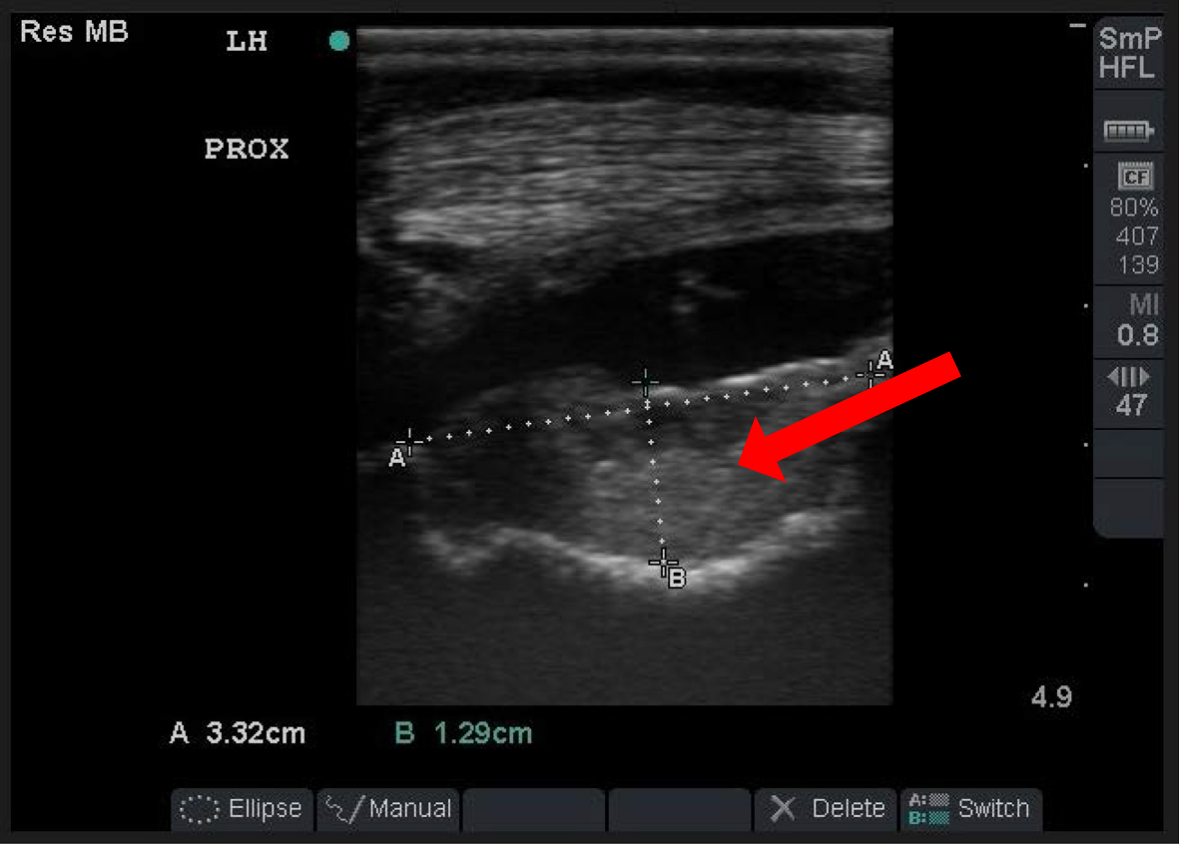
Image of ultrasound of horse stifle taken one month post implantation of biomaterial scaffold (arrow) into naturally occurring osteochondral defect of trochlea of femur. This demonstrates the large defects occurring in the horse. The ability to image the implanted scaffold during the post-operative period is also a significant advantage to the large animal model

Horse (equine) models offer several advantages in the investigation of cartilage repair strategies. Horses are animals primarily bred and kept for their athletic performance and, as a result, suffer regularly from cartilage injuries and joint diseases such as osteoarthritis and osteochondrosis. Hence performing pre-clinical evaluations on cartilage repair mechanisms may be of direct benefit to the species itself (Fig. 3.) (Malda et al. 2012). However candidates for entry to a study must be screened in advance for naturally occurring disease to avoid affecting results. Due to the large joint surface, arthroscopies are routine and can be used both for cartilage defect creation and repair, and longitudinal follow up on the process of cartilage repair at different time points. The equine model has critical size defects up to 9 mm, cartilage mean thickness of 2.0–3.0 mm and a vertically loaded stifle joint during gait, and so is beneficial for translatable cartilage studies and especially partial thickness defects which are the most relevant to human therapy (ASTM F2451–05 2010; Ahern et al. 2009; Malda et al. 2012). The horse is the largest animal model in use as a model for cartilage repair, commonly weighing around 500–600 kg, the joint is, therefore, adapted to withstand elevated loads, with a hardened subchondral bone and efficient joint force distribution (Chu et al. 2010a). Bone mineral density in the horse MFC is reportedly similar to that in humans at the bone plate (1.19 g/cm^3^) but higher at a depth 3 mm (0.64 g/cm^3^). Bone mineral density values were found to be similar to humans in the horse lateral trochlea at a depth of 3 mm (0.5 g/cm^3^) (Chevrier et al. 2015). Bone volume fraction is reportedly higher in horse than in humans in the MFC (47 ± 8 %) at a depth and 3 mm. (Chevrier et al. 2015) Loading is of concern as continuous static loading of weight bearing condyles of the joint cannot be minimised, as a result the lateral trochlea of the femur where loading is intermittent is the most common location for cartilage defects (Ahern et al. 2009). In the horse model it is also common to use the carpus and the tibiotarsal joints and joints of the middle carpal bones for defect formation, as chondral injuries can be common here and the stifle joint is difficult to access for diagnostic imaging such as MRI due to the bulk of the upper hind limb. The stifle is however amenable to ultrasound in the hands of an experienced user (Fig. 4) (Hurtig et al. 2011; Ahern et al. 2009; Hurtig et al. 2001; Vachon et al. 1992; Rautiainen et al. 2013). While the horse is an appealing model in terms of cartilage thickness and joint morphology, a highly specialized and a well-equipped centre with well-trained personnel is required to carry out equine surgeries, and they require a large specialised habitat. These all lead to substantially increased costs involved in the study. In addition to practical considerations, horses are also subject to stringent licencing in some jurisdictions due to their historic status as a companion animal (Hurtig et al. 2011; Animals (Scientific Procedures) Act. Sect. 14 1986).

## Operative factors influencing model selection

There are many reasons to choose one model over another (Table 2.), however surgical limitations of certain models play a large part in the selection of the appropriate group. Size is one of the main factors. In order to implant a biomaterial in a particular area the area must be of sufficient size to allow implantation. For example the knee joint of a rat has a cartilage thickness of 0.1 mm on the MFC, and in the mouse cartilage is only a few cell layers thick (Chu et al. 2010a) compared to between 0.8 and 2.0 mm on the MFC of the caprine stifle (ASTM F2451–05 2010; Chu et al. 2010a). To create a defect this small presents a technical difficulty for the operator meaning purely chondral defects are impossible and can lead to large inter-animal variation even when producing osteochondral defects meaning a much greater sample size would have to be used to achieve a statistically significant outcome contravening the 3 R’s (Russell & Burch 1959).

**Table 2:** Advantages and disadvantages of various in vivo models commonly used in the assessment of biomaterial strategies for cartilage defect repair

Species	Advantage	Disadvantage
Mouse	Low cost, manageable easily available Transgenic and athymic strains available Can be used in subcutaneous and intramuscular model for degradation rate and safety profile	Very small joints–in situ examination impossible
Rat	Low cost, easily available Athymic strains available Maintain in-house	Permanently open growth plates accelerating intrinsic healing Increased density of cells in cartilage causing more efficient healing Partial thickness defects impossible
Rabbit	Low cost Maintain in-house	Increased intrinsic healing due to increased cell density Very different load characteristics Consistent partial thickness defects very difficult to achieve
Dog	Naturally occurring disease state Co-operate with rehabilitation regime	Thin cartilage Small critical size defect (4 mm) Complex ethical approval process
Pig	Biochemistry similar to humans Bone apposition rate and trabecular thickness similar to human Partial thickness defects possible	Expensive Difficult to obtain at skeletal maturity Specialised habitat Temperament
Goat	Anatomy and biomechanics similar to humans Partial thickness defects possible Easily available Low maintenance	Subchondral cyst formation
Sheep	Anatomy similar to humans Partial thickness defects possible Easily available Low maintenance	Subchondral cyst formation
Horse	Large defects similar to humans Partial thickness large diameter defects possible Naturally occurring defects Similar biomechanics in trochlear groove Second look arthroscopy possible	Expensive to acquire and maintain – specialised centre required Cannot avoid weight bearing on the joint during rehab phase if required Very dense subchondral bone MRI/CT impossible due to size

Post-operative rehabilitation of cartilage repair in humans varies from centre to centre, it has been shown free movement and gradual increase in weight bearing improves outcomes, however, this is complex to ensure in animal models (Nishino et al. 2010; Assche et al. 2011). When using an in vivo model casts can be used to immobilise rat limbs (Kojima et al. 2014; Maldonado et al. 2013), however it is more common in large animals such as goats and sheep to keep them in small pens in the immediate post-operative period (Marmotti et al. 2013) to limit their mobilising. In some animals, such as horses, non-weight bearing is impossible, and can cause severe life-threatening illnesses. Horses will fully weight bear immediately post recovery, but if the defect area is on the patellofemoral joint rather than the tibiofemoral joint, a regimen of supervised walking building up to limited running can offload the patellofemoral joint in the post-operative phase.

## Ex vivo factors influencing model selection

The chosen model affects the analysis that can the carried out, both during the study and post euthanasia at the study end point, and also the results that can be obtained. In smaller animals, it is possible to do in situ microcomputed tomography or magnetic resonance imaging on live animals, allowing good radiological scoring. Due to limited equipment availability this is more challenging for large animals. In large animals, the larger joint size allows for second look arthroscopy to be carried out during of the study. This can provide useful information about the repair tissue prior to the study endpoint. The use of large animal models results in larger tissue specimens for analysis. For example, in the horse model, the critical size defect is 9 mm. This poses some technical disadvantages as the dense subchondral bone requires longer decalcification times prior to histological staining. However, division of samples is possible without much difficulty, allowing for example, mechanical testing to be performed on one half and histological staining on the other. This doubles the amount of information collected from the experiment. Dividing a 6 mm (caprine) or 7 mm (ovine) can be a more daunting prospect and can leave artefact obscuring true results. These factors must be carefully considered at the outset of the study and the appropriate model chosen for the results and analyses required.

## Conclusion

There is a significant pre-clinical gap to be bridged in the development of a device to ease suffering and halt joint degeneration before it can be used as a therapeutic clinically. Therefore, the selection of an appropriate pre-clinical in vivo model is important in ensuring successful translation to the clinic. The financial and labour costs involved in a large animal study can be prohibitive, and so for a proof of concept or degradation and safety profile it can be appropriate to use a small animal or rodent model before confirming effectiveness in a large animal study. It is also important to consider the different biomechanics and biokinematics of joints in quadrupeds and how the contact pressures on the weight bearing areas of the joint are affected along with stresses and strains on areas of joints not completely analogous to stresses and strains of the human knee. Therefore, in many ways, the caprine model is the most appropriate model for large scale large animal studies in cartilage surface defect repair, as the anatomy is closest to humans, they have similar biomechanics of their stifle joint to human knees, they have an adequate cartilage thickness allowing for partial and full thickness defects. Goats do not require specialised housing other than warm indoor bedding in winter and access to pasture in summer. In addition, goats are widely available as they are commonly used in agriculture. Pre-clinical studies are important to ensure safety and efficacy of biomaterials prior to widespread use, however, there are significant differences in the anatomy and biomechanics of different animal models and of humans. In order to enable the successful translation of biomaterials to the clinic, these differences must be recognised and considered in both study design and in comparing study outcomes.

## References

[CR1] Ahern BJ, Parvizi J, Boston R, Schaer TP (2009). Preclinical animal models in single site cartilage defect testing: a systematic review. Osteoarthritis Cartilage.

[CR2] Allen MJ, Houlton JE, Adams SB, Rushton N (1998). The surgical anatomy of the stifle joint in sheep. Vet Surg.

[CR3] Almeida HV, Cunniffe GM, Vinardell T, Buckley CT, O'Brien FJ, Kelly DJ (2015). Coupling Freshly Isolated CD44 Infrapatellar Fat Pad-Derived Stromal Cells with a TGF-beta3 Eluting Cartilage ECM-Derived Scaffold as a Single-Stage Strategy for Promoting Chondrogenesis. Adv Healthc Mater.

[CR4] An YH, Freidman RJ (1999). Animal Models in Orthopaedic Research.

[CR5] Animals (Scientific Procedures) Act. Sect. 14 (1986).

[CR6] Assche DV, Caspel DV, Staes F, Saris DB, Bellemans J, Vanlauwe J (2011). Implementing one standardized rehabilitation protocol following autologous chondrocyte implantation or microfracture in the knee results in comparable physical therapy management. Physiother Theory Pract.

[CR7] ASTM F2451–05 (2010). Standard Guide for in vivo Assessment of Implantable Devices Intended to Repair or Regenerate Articular Cartilage.

[CR8] Boopalan PR, Arumugam S, Livingston A, Mohanty M, Chittaranjan S (2011). Pulsed electromagnetic field therapy results in healing of full thickness articular cartilage defect. Int Orthop.

[CR9] Brehm W, Aklin B, Yamashita T, Rieser F, Trub T, Jakob RP (2006). Repair of superficial osteochondral defects with an autologous scaffold-free cartilage construct in a caprine model: implantation method and short-term results. Osteoarthritis Cartilage.

[CR10] Breinan HA, Martin SD, Hsu HP, Spector M (2000). Healing of canine articular cartilage defects treated with microfracture, a type-II collagen matrix, or cultured autologous chondrocytes. J Orthop Res.

[CR11] Brittberg M, Lindahl A, Nilsson A, Ohlsson C, Isaksson O, Peterson L (1994). Treatment of deep cartilage defects in the knee with autologous chondrocyte transplantation. N Engl J Med.

[CR12] Brittberg M, Sjogren-Jansson E, Lindahl A, Peterson L (1997). Influence of fibrin sealant (Tisseel) on osteochondral defect repair in the rabbit knee. Biomaterials.

[CR13] Camp CL, Stuart MJ, Krych AJ (2014). Current concepts of articular cartilage restoration techniques in the knee. Sports health.

[CR14] Chevrier A, Kouao AS, Picard G, Hurtig MB, Buschmann MD (2015). Interspecies comparison of subchondral bone properties important for cartilage repair. J Orthop Res.

[CR15] Choi B, Kim S, Fan J, Kowalski T, Petrigliano F, Evseenko D (2015). Covalently conjugated transforming growth factor-beta1 in modular chitosan hydrogels for the effective treatment of articular cartilage defects. Biomaterials science.

[CR16] Christensen BB, Foldager CB, Olesen ML, Vingtoft L, Rölfing JHD, Ringgaard S (2015). Experimental articular cartilage repair in the Göttingen minipig: the influence of multiple defects per knee. J Exp Orthop.

[CR17] Chu CR (2001). Chondral and osteochondral injuries: mechanisms of injury and repair responses. Oper Tech Orthop.

[CR18] Chu CR, Dounchis JS, Yoshioka M, Sah RL, Coutts RD, Amiel D (1997). Osteochondral repair using perichondrial cells. A 1-year study in rabbits. Clin Orthop Relat Res.

[CR19] Chu CR, Szczodry M, Bruno S (2010). Animal models for cartilage regeneration and repair. Tissue Eng B Rev.

[CR20] Chu CR, Szczodry M, Bruno S (2010). Animal models for cartilage regeneration and repair. Tissue Eng Part B-Re.

[CR21] Chung JY, Song M, Ha CW, Kim JA, Lee CH, Park YB (2014). Comparison of articular cartilage repair with different hydrogel-human umbilical cord blood-derived mesenchymal stem cell composites in a rat model. Stem Cell Res Ther.

[CR22] Cook JL, Hung CT, Kuroki K, Stoker AM, Cook CR, Pfeiffer FM (2014). Animal models of cartilage repair. Bone Joint Res.

[CR23] Dahlin RL, Kinard LA, Lam J, Needham CJ, Lu S, Kasper FK (2014). Articular chondrocytes and mesenchymal stem cells seeded on biodegradable scaffolds for the repair of cartilage in a rat osteochondral defect model. Biomaterials.

[CR24] Ebert JR, Robertson WB, Lloyd DG, Zheng MH, Wood DJ, Ackland T (2008). Traditional vs accelerated approaches to post-operative rehabilitation following matrix-induced autologous chondrocyte implantation (MACI): comparison of clinical, biomechanical and radiographic outcomes. Osteoarthritis Cartilage.

[CR25] Ebihara G, Sato M, Yamato M, Mitani G, Kutsuna T, Nagai T (2012). Cartilage repair in transplanted scaffold-free chondrocyte sheets using a minipig model. Biomaterials.

[CR26] Engkvist O (1979). Reconstruction of patellar articular cartilage with free autologous perichondrial grafts. An experimental study in dogs. Scand J Plast Reconstr Surg.

[CR27] Erggelet C, Endres M, Neumann K, Morawietz L, Ringe J, Haberstroh K (2009). Formation of cartilage repair tissue in articular cartilage defects pretreated with microfracture and covered with cell-free polymer-based implants. J Orthop Res.

[CR28] Etterlin PE, Ytrehus B, Lundeheim N, Heldmer E, Osterberg J, Ekman S (2014). Effects of free-range and confined housing on joint health in a herd of fattening pigs. BMC Vet Res.

[CR29] Fisher MB, Belkin NS, Milby AH, Henning EA, Bostrom M, Kim M (2015). Cartilage repair and subchondral bone remodeling in response to focal lesions in a mini-pig model: implications for tissue engineering. Tissue Eng A.

[CR30] Fragonas E, Valente M, Pozzi-Mucelli M, Toffanin R, Rizzo R, Silvestri F (2000). Articular cartilage repair in rabbits by using suspensions of allogenic chondrocytes in alginate. Biomaterials.

[CR31] Frisbie DD, Lu Y, Kawcak CE, DiCarlo EF, Binette F, McIlwraith CW (2009). In vivo evaluation of autologous cartilage fragment-loaded scaffolds implanted into equine articular defects and compared with autologous chondrocyte implantation. Am J Sports Med.

[CR32] Funayama A, Niki Y, Matsumoto H, Maeno S, Yatabe T, Morioka H (2008). Repair of full-thickness articular cartilage defects using injectable type II collagen gel embedded with cultured chondrocytes in a rabbit model. J Orthop Sci.

[CR33] Gelse K, von der Mark K, Aigner T, Park J, Schneider H (2003). Articular cartilage repair by gene therapy using growth factor-producing mesenchymal cells. Arthritis Rheum.

[CR34] Getgood AM, Kew SJ, Brooks R, Aberman H, Simon T, Lynn AK (2012). Evaluation of early-stage osteochondral defect repair using a biphasic scaffold based on a collagen-glycosaminoglycan biopolymer in a caprine model. Knee.

[CR35] Gollehon DL, Torzilli PA, Warren RF (1987). The role of the posterolateral and cruciate ligaments in the stability of the human knee. A biomechanical study. J Bone Joint Sur Am.

[CR36] Guo X, Wang C, Zhang Y, Xia R, Hu M, Duan C (2004). Repair of large articular cartilage defects with implants of autologous mesenchymal stem cells seeded into beta-tricalcium phosphate in a sheep model. Tissue Eng.

[CR37] Gushue DL, Houck J, Lerner AL (2005). Rabbit knee joint biomechanics: motion analysis and modeling of forces during hopping. J Orthop Res.

[CR38] Hangody L, Feczko P, Bartha L, Bodo G, Kish G (2001). Mosaicplasty for the treatment of articular defects of the knee and ankle. Clin Orthop Relat Res.

[CR39] Hendrickson DA, Nixon AJ, Grande DA, Todhunter RJ, Minor RM, Erb H (1994). Chondrocyte-fibrin matrix transplants for resurfacing extensive articular cartilage defects. J Orthop Res.

[CR40] Hoemann CD, Hurtig M, Rossomacha E, Sun J, Chevrier A, Shive MS (2005). Chitosan-glycerol phosphate/blood implants improve hyaline cartilage repair in ovine microfracture defects. J Bone Joint Sur Am.

[CR41] Hoemann C, Kandel R, Roberts S, Saris DB, Creemers L, Mainil-Varlet P (2011). International Cartilage Repair Society (ICRS) recommended guidelines for histological endpoints for cartilage repair studies in animal models and clinical trials. Cartilage.

[CR42] Hunziker EB (1999). Biologic repair of articular cartilage. Defect models in experimental animals and matrix requirements. Clin Orthop Relat Res.

[CR43] Hunziker EB, Driesang IM, Morris EA (2001) Chondrogenesis in cartilage repair is induced by members of the transforming growth factor-beta superfamily. Clin Orthop Relat Res 391 Suppl:S171-81 10.1097/00003086-200110001-00017 11603702

[CR44] Hunziker EB, Quinn TM, Hauselmann HJ (2002). Quantitative structural organization of normal adult human articular cartilage. Osteoarthritis Cartilage.

[CR45] Hunziker EB, Lippuner K, Keel MJ, Shintani N (2015). An educational review of cartilage repair: precepts & practice - myths & misconceptions - progress & prospects. Osteoarthritis Cartilage.

[CR46] Hurtig M, Pearce S, Warren S, Kalra M, Miniaci A (2001). Arthroscopic mosaic arthroplasty in the equine third carpal bone. Veterinary surgery : VS.

[CR47] Hurtig MB, Buschmann MD, Fortier LA, Hoemann CD, Hunziker EB, Jurvelin JS (2011). Preclinical studies for cartilage repair: recommendations from the international cartilage repair society. Cartilage.

[CR48] Igarashi T, Iwasaki N, Kawamura D, Kasahara Y, Tsukuda Y, Ohzawa N (2012). Repair of articular cartilage defects with a novel injectable in situ forming material in a canine model. J Biomed Mater Res A.

[CR49] Jackson DW, Lalor PA, Aberman HM, Simon TM (2001). Spontaneous repair of full-thickness defects of articular cartilage in a goat model. A preliminary study. J Bone Joint Sur Am.

[CR50] Jiang CC, Chiang H, Liao CJ, Lin YJ, Kuo TF, Shieh CS (2007). Repair of porcine articular cartilage defect with a biphasic osteochondral composite. J Orthop Res.

[CR51] Jurgens WJ, Kroeze RJ, Zandieh-Doulabi B, van Dijk A, Renders GA, Smit TH (2013). One-step surgical procedure for the treatment of osteochondral defects with adipose-derived stem cells in a caprine knee defect: a pilot study. Bio Research open access.

[CR52] Klein TJ, Malda J, Sah RL, Hutmacher DW (2009). Tissue engineering of articular cartilage with biomimetic zones. Tissue Eng B Rev.

[CR53] Kojima S, Hoso M, Watanabe M, Matsuzaki T, Hibino I, Sasaki K (2014). Experimental joint immobilization and remobilization in the rats. J Phys Ther Sci.

[CR54] Kon E, Delcogliano M, Filardo G, Fini M, Giavaresi G, Francioli S (2010). Orderly osteochondral regeneration in a sheep model using a novel nano-composite multilayered biomaterial. J Orthop Res.

[CR55] Kon E, Mutini A, Arcangeli E, Delcogliano M, Filardo G, Nicoli Aldini N (2010). Novel nanostructured scaffold for osteochondral regeneration: pilot study in horses. J Tissue Eng Regen Med.

[CR56] Kuroda R, Usas A, Kubo S, Corsi K, Peng H, Rose T (2006). Cartilage repair using bone morphogenetic protein 4 and muscle-derived stem cells. Arthritis Rheum.

[CR57] LaPrade RF, Kimber KA, Wentorf FA, Olson EJ (2006). Anatomy of the posterolateral aspect of the goat knee. J Orthop Res.

[CR58] Levingstone TJ, Matsiko A, Dickson GR, O’Brien FJ, Gleeson JP (2014) A biomimetic multi-layered collagen-based scaffold for osteochondral repair. Acta Biomater 10(5):1996–2004 10.1016/j.actbio.2014.01.005 24418437

[CR59] Levingstone TJ, Thompson E, Matsiko A, Schepens A, Gleeson JP, O’Brien FJ (2015) Multi-layered collagen-based scaffolds for osteochondral defect repair in rabbits. Acta Biomater In Press. 10.1016/j.actbio.2015.12.034 26724503

[CR60] Libbin RM, Rivera ME (1989). Regeneration of growth plate cartilage induced in the neonatal rat hindlimb by reamputation. J Orthop Res.

[CR61] Lohan A, Marzahn U, El Sayed K, Bock C, Haisch A, Kohl B (2013). Heterotopic and orthotopic autologous chondrocyte implantation using a minipig chondral defect model. Ann Anat.

[CR62] Lu Y, Dhanaraj S, Wang Z, Bradley DM, Bowman SM, Cole BJ (2006). Minced cartilage without cell culture serves as an effective intraoperative cell source for cartilage repair. J Orthop Res.

[CR63] Luengo Gimeno F, Gatto S, Ferro J, Croxatto JO, Gallo JE (2006). Preparation of platelet-rich plasma as a tissue adhesive for experimental transplantation in rabbits. Thromb J.

[CR64] Madry H, Ochi M, Cucchiarini M, Pape D, Seil R (2015). Large animal models in experimental knee sports surgery: focus on clinical translation. J Exp Orthop.

[CR65] Malda J, Benders KE, Klein TJ, de Grauw JC, Kik MJ, Hutmacher DW (2012). Comparative study of depth-dependent characteristics of equine and human osteochondral tissue from the medial and lateral femoral condyles. Osteoarthritis Cartilage.

[CR66] Maldonado DC, Silva MC, Neto Sel R, de Souza MR, de Souza RR (2013). The effects of joint immobilization on articular cartilage of the knee in previously exercised rats. J Anat.

[CR67] Mankin HJ (1982). The response of articular cartilage to mechanical injury. J Bone Joint Surg Am.

[CR68] Marmotti A, Bruzzone M, Bonasia DE, Castoldi F, Rossi R, Piras L (2012). One-step osteochondral repair with cartilage fragments in a composite scaffold. Knee Surg Sports Traumatol Arthrosc.

[CR69] Marmotti A, Bruzzone M, Bonasia DE, Castoldi F, Von Degerfeld MM, Bignardi C (2013). Autologous cartilage fragments in a composite scaffold for one stage osteochondral repair in a goat model. Eur Cell Mater.

[CR70] Maruyama Y (1979). An experimental study on cartilage foramation in autogenous perichondrial transplantation in rabbits. Keio J Med.

[CR71] Messner K, Fahlgren A, Persliden J, Andersson BM (2001). Radiographic joint space narrowing and histologic changes in a rabbit meniscectomy model of early knee osteoarthrosis. Am J Sports Med.

[CR72] Milano G, Sanna Passino E, Deriu L, Careddu G, Manunta L, Manunta A (2010). The effect of platelet rich plasma combined with microfractures on the treatment of chondral defects: an experimental study in a sheep model. Osteoarthritis Cartilage.

[CR73] Nishino T, Ishii T, Chang F, Yanai T, Watanabe A, Ogawa T (2010). Effect of gradual weight-bearing on regenerated articular cartilage after joint distraction and motion in a rabbit model. J Orthop Res.

[CR74] Nixon AJ, Rickey E, Butler TJ, Scimeca MS, Moran N, Matthews GL (2015). A chondrocyte infiltrated collagen type I/III membrane (MACI((R)) implant) improves cartilage healing in the equine patellofemoral joint model. Osteoarthritis Cartilage.

[CR75] Nordling C, Karlsson-Parra A, Jansson L, Holmdahl R, Klareskog L (1992). Characterization of a spontaneously occurring arthritis in male DBA/1 mice. Arthritis Rheum.

[CR76] Nukavarapu SP, Dorcemus DL (2013). Osteochondral tissue engineering: current strategies and challenges. Biotechnol Adv.

[CR77] Orth P, Goebel L, Wolfram U, Ong MF, Graber S, Kohn D (2012). Effect of subchondral drilling on the microarchitecture of subchondral bone: analysis in a large animal model at 6 months. Am J Sports Med.

[CR78] Orth P, Meyer HL, Goebel L, Eldracher M, Ong MF, Cucchiarini M (2013). Improved repair of chondral and osteochondral defects in the ovine trochlea compared with the medial condyle. J Orthop Res.

[CR79] Orth P, Zurakowski D, Alini M, Cucchiarini M, Madry H (2013). Reduction of sample size requirements by bilateral versus unilateral research designs in animal models for cartilage tissue engineering. Tissue Eng Part C, Methods.

[CR80] Pallante-Kichura AL, Cory E, Bugbee WD, Sah RL (2013). Bone cysts after osteochondral allograft repair of cartilage defects in goats suggest abnormal interaction between subchondral bone and overlying synovial joint tissues. Bone.

[CR81] Patil S, Steklov N, Song L, Bae WC, D’Lima DD (2014). Comparative biomechanical analysis of human and caprine knee articular cartilage. Knee.

[CR82] Peterson L, Minas T, Brittberg M, Lindahl A (2003). Treatment of osteochondritis dissecans of the knee with autologous chondrocyte transplantation: results at two to ten years. J Bone Joint Sur Am.

[CR83] Proffen BL, McElfresh M, Fleming BC, Murray MM (2012). A comparative anatomical study of the human knee and six animal species. Knee.

[CR84] Rautiainen J, Lehto LJ, Tiitu V, Kiekara O, Pulkkinen H, Brunott A (2013). Osteochondral repair: evaluation with sweep imaging with fourier transform in an equine model. Radiology.

[CR85] Roth JH, Mendenhall HV, McPherson GK (1988). The effect of immobilization on goat knees following reconstruction of the anterior cruciate ligament. Clin Orthop Relat Res.

[CR86] Russell WMS, Burch RL (1959). The Principles of Humane Experimental Technique.

[CR87] Schneider U, Schmidt-Rohlfing B, Gavenis K, Maus U, Mueller-Rath R, Andereya S (2011). A comparative study of 3 different cartilage repair techniques. Knee Surg Sports Traumatol Arthrosc.

[CR88] Shahgaldi BF (1998). Repair of large osteochondral defects: load-bearing and structural properties of osteochondral repair tissue. Knee.

[CR89] Shortkroff S, Barone L, Hsu HP, Wrenn C, Gagne T, Chi T (1996). Healing of chondral and osteochondral defects in a canine model: the role of cultured chondrocytes in regeneration of articular cartilage. Biomaterials.

[CR90] Singh NK, Singh GR, Jeong DK, Lee SJ (2013). Healing of full-thickness articular cartilage defects treated with cultured autologous chondrogenic satellite cells isolated from chondral stem cell niche in rabbits. J Surg Res.

[CR91] Steadman JR, Briggs KK, Rodrigo JJ, Kocher MS, Gill TJ, Rodkey WG (2003). Outcomes of microfracture for traumatic chondral defects of the knee: average 11-year follow-up. Arthroscopy.

[CR92] Taylor WR, Heller MO, Bergmann G, Duda GN (2004). Tibio-femoral loading during human gait and stair climbing. J Orthop Res.

[CR93] Taylor WR, Ehrig RM, Heller MO, Schell H, Seebeck P, Duda GN (2006). Tibio-femoral joint contact forces in sheep. J Biomech.

[CR94] Vachon AM, McIlwraith CW, Powers BE, McFadden PR, Amiel D (1992). Morphologic and biochemical study of sternal cartilage autografts for resurfacing induced osteochondral defects in horses. Am J Vet Res.

[CR95] Vogt S, Angele P, Arnold M, Brehme K, Cotic M, Haasper C (2013). Practice in rehabilitation after cartilage therapy: an expert survey. Arch Orthop Trauma Surg.

[CR96] von Rechenberg B, Akens MK, Nadler D, Bittmann P, Zlinszky K, Kutter A (2003). Changes in subchondral bone in cartilage resurfacing--an experimental study in sheep using different types of osteochondral grafts. Osteoarthritis Cartilage.

[CR97] Wang DA, Varghese S, Sharma B, Strehin I, Fermanian S, Gorham J (2007). Multifunctional chondroitin sulphate for cartilage tissue-biomaterial integration. Nat Mater.

[CR98] Wei X, Rasanen T, Messner K (1998). Maturation-related compressive properties of rabbit knee articular cartilage and volume fraction of subchondral tissue. Osteoarthritis Cartilage.

[CR99] Wolfensohn S, Lloyd M (2003). Handbook of Laboratory Animal Management and Welfare.

[CR100] Yanai T, Ishii T, Chang F, Ochiai N (2005). Repair of large full-thickness articular cartilage defects in the rabbit: the effects of joint distraction and autologous bone-marrow-derived mesenchymal cell transplantation. J Bone Joint Sur British.

